# Effect of a Low Dose of Carvedilol on Cyclophosphamide-Induced Urinary Toxicity in Rats—A Comparison with Mesna

**DOI:** 10.3390/ph14121237

**Published:** 2021-11-29

**Authors:** Anna Merwid-Ląd, Piotr Ziółkowski, Marta Szandruk-Bender, Agnieszka Matuszewska, Adam Szeląg, Małgorzata Trocha

**Affiliations:** 1 Department of Pharmacology, Wroclaw Medical University, J. Mikulicza-Radeckiego 2, 50-345 Wrocław, Poland; agnieszka.matuszewska@umw.edu.pl (A.M.); adam.szelag@umw.edu.pl (A.S.); malgorzata.trocha@umw.edu.pl (M.T.); 2 Department of Pathology, Wroclaw Medical University, K. Marcinkowskiego 1, 50-368 Wrocław, Poland; piotr.ziolkowski@umw.edu.pl

**Keywords:** cyclophosphamide, carvedilol, urinary bladder, kidney, oxidative stress, toxicity

## Abstract

One of the major side effects of cyclophosphamide (CPX)—an alkylating anticancer drug that is still clinically used—is urotoxicity with hemorrhagic cystitis. The present study was designed to evaluate the ability of carvedilol to protect rats from cyclophosphamide-induced urotoxicity. Rats were injected intraperitoneally (*i.p.*) with CPX (200 mg/kg) and administered carvedilol (2 mg/kg) intragastrically a day before, at the day and a day after a single *i.p*. injection of CPX, with or without mesna (40, 80, and 80 mg/kg *i.p.* 20 min before, 4 h and 8 h after CPX administration, respectively). Pretreatment with carvedilol partly prevented the CPX-induced increase in urinary bladder and kidney index, and completely protects from CPX-evoked alterations in serum potassium and creatinine level, but did not prevent histological alterations in the urinary bladder and hematuria. However, carvedilol administration resulted in significant restoration of kidney glutathione (GSH) level and a decrease in kidney interleukin 1β (IL-1β) and plasma asymmetric dimethylarginine (ADMA) concentrations. Not only did mesna improve kidney function, but it also completely reversed histological abnormalities in bladders and prevented hematuria. In most cases, no significant interaction of carvedilol with mesna was observed, although the effect of both drugs together was better than mesna given alone regarding plasma ADMA level and kidney IL-1β concentration. In conclusion, carvedilol did not counteract the injury caused in the urinary bladders but restored kidney function, presumably via its antioxidant and anti-inflammatory properties.

## 1. Introduction

Many drugs, excreted with the kidneys, are later accumulated in urine in high amounts and may interact with urothelium for a long time, especially in the urinary bladder during the night. Urothelium, lining the urinary tract from the kidney to the urethra, is a high-resistance barrier preventing the diffusion of a variety of substances excreted by kidneys to the tissues. This specific structure is, therefore, exposed to high concentrations of endogenous waste products as well as some xenobiotics, including drugs. Additionally, the urothelium is exposed to mechanical stretch due to the physiology of the urination process [1]. Despite the high resistance of urothelium to noxious stimuli, they may irritate the urinary tract, and trigger alterations in the urothelium, nonbacterial inflammatory response (cystitis) leading finally to various injuries, e.g., edema, erosions, ulcerations, or even hematuria. Although damages in the urinary tract are the best described for systemic cyclophosphamide (CPX) administration, some other anticancer agents may interact with urothelium, causing chemical cystitis as it was found in the case of thiotepa, mitomycin C, doxorubicin, or epirubicin, especially when they are given intravesically for urinary bladder cancer treatment [2,3].

Despite many new, targeted biologic therapies in the treatment of malignancies or autoimmune diseases [4], conventional drugs, such as cyclophosphamide, are still in clinical use. Cyclophosphamide is a DNA-alkylating agent registered, among others, in the treatment of acute and chronic leukemias, Hodgkin’s and non-Hodgkin’s lymphomas, breast and lung cancers in adults as well as sarcomas in children. It may be also used in some life-threatening autoimmune diseases [2]. There are many possible adverse effects of CPX, very problematic for the patients, and sometimes even fatal in the outcome, and among these are the disorders of the urinary tract with very common cystitis or less common hemorrhagic cystitis with micro- or macroscopic hematuria. Pyelitis, injury of renal tubules, and increased serum creatinine levels with kidney failure are also described. Toxicity in the urinary tract may be seen after both long- and short-term treatment. Ulcerations, necrosis, fibrosis, contracture, or sometimes secondary cancers are also possible complications in the urinary bladder [2,5].

The mechanism of CPX-induced injury in the urinary tract is multifactorial and triggered mainly by the interaction of acrolein, a toxic CPX metabolite, with urothelium that induces the inflammatory response. Up-regulation of nuclear factor kappa B (NF-κB) is important in causing such cytokines to release as tumor necrosis factor-alpha (TNF-α), interleukin 1β (IL-1β), or IL-6. Additionally, the generation of reactive oxygen species (ROS), overexpression of cyclooxygenase-2 (COX-2) and inducible nitric oxide synthase (iNOS), involvement of bradykinin, substance P, and platelet-activating factor (PAF) is important or suggested in this pathological pathway. This leads to the destruction of the urothelium, erosions, ulcerations, pain, and bladder overactivity with the possible role of activation of alpha-1 adrenergic receptors [6,7,8,9]. In addition to the well-described toxicity of CPX in urinary bladders, kidneys are also often affected. Changes in renal function, urine amount, and composition or kidney morphology were noticed [10,11].

For the prevention of CPX-induced hemorrhagic cystitis and reduction in urinary toxicity, bladder irrigation or forced diuresis with prior hydration with or without intravenous administration of mesna (sodium 2-mercapto-ethanesulphonate) are commonly used. The postulated mechanism of mesna, a sulfhydryl compound, is that it binds the methyl group of acrolein which leads to the formation of thioether, which is non-toxic, and does not trigger the inflammatory process [12,13]. Recently, the efficacy of mesna in clinical practice has been widely discussed and some trials, as well as the literature overview, indicate great differences in the conclusions drawn [14,15,16]. Therefore, there is still a great need to look for new compounds, which may prevent CPX-induced cystitis decreasing the risk of severe hemorrhagic outcomes and additionally may prevent kidney injury.

Carvedilol is a highly lipophilic, third-generation nonselective beta-adrenergic antagonist, additionally acting as an antagonist of alpha-1 receptors with a ratio of beta to alpha blockade of about 10:1. It is also one of the beta adrenolytic drugs described as biased agonists promoting β-arrestin-mediated ERK phosphorylation, which finally suggests inhibition of neoplastic transformation and carcinogenesis [17,18]. 

It is postulated that carvedilol, and its metabolites, are potent antioxidants and scavengers of ROS [19,20]. It is worth mentioning that carvedilol may decrease levels of proinflammatory cytokines, e.g., IL-1β, TNF-α and inhibits COX-2, matrix metalloproteinase 2 (MMP-2), or NF-κB expression in various inflammatory models [21,22,23].

Many studies focused on the preventive potential of carvedilol in anthracycline-induced cardiotoxicity in humans and in a variety of in vitro or animal models [20,24,25], but it was also found that carvedilol may prevent, e.g., from cisplatin-induced kidney injury [26] or testicular and sperm damages [27], may exert nephroprotective action in the model of cyclosporine-induced nephrotoxicity [28] or may prevent from gastric mucosa injury after administration of acetylsalicylic acid [29]. In addition to the postulated protection in different models of tissue damages, carvedilol seems to modulate MDR1-mediated resistance to anticancer agents [30].

Based on these premises, our study aimed to evaluate the potential of carvedilol in acute, cyclophosphamide-induced urinary tract toxicity. 

## 2. Results

### 2.1. Body Weight Changes and Organ Weight

**Figure 1:**
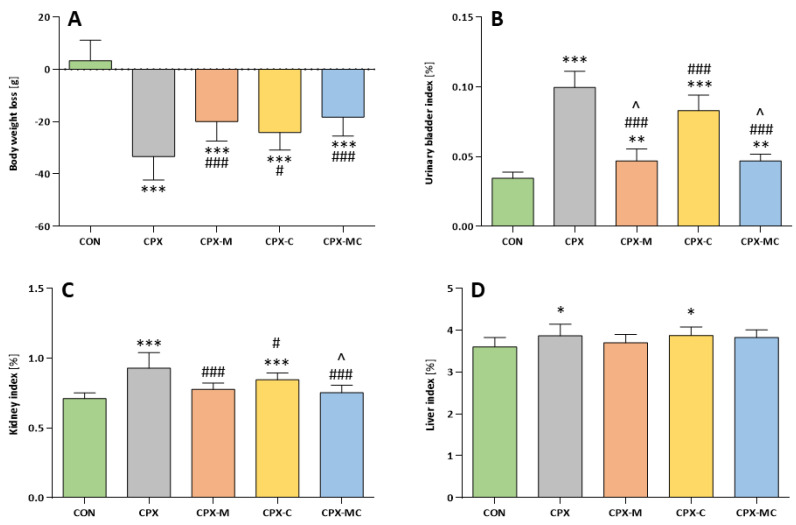
Body weight loss (**A**), urinary bladder index (**B**), kidney index (**C**), and liver index (**D**) calculated as percentages of the total body weight. Data are presented as means ± SD. A detailed description of experimental groups is presented in Figure 1 and the Materials and Methods section. * *p* < 0.05, ** *p* < 0.01, *** *p* < 0.001 vs. CON group; # *p* < 0.05, ### *p* < 0.001 vs. CPX group; ^ *p* < 0.05 vs. CPX-C group.

After the adaptation period, on day 1 of the main experiment, the mean body weight in all experimental groups was comparable and no significant differences between groups were noticed (CON—317.5 ± 25.3 g, CPX—324.2 ± 22.7 g, CPX-M—316.7 ± 8.9 g, CPX-C—304.2 ± 10.8, CPX-MC—315.8 ± 12.4 g). For an explanation of the groups’ abbreviations, please see the Materials and Methods section. In all groups receiving cyclophosphamide intraperitoneally in a single dose of 200 mg/kg, a significant decrease in body weight in comparison to the control group was found with the largest body weight loss in the group receiving only CPX. Administration of mesna (200 mg/kg in divided doses), alone or with carvedilol (2 mg/kg, for 3 days), partly prevented the body weight decrease (significant differences vs. CON and CPX group). The action was the least pronounced in the CPX-C group (Figure 1A). 

The urinary bladder, kidney, and liver indexes are presented in Figure 1B–D, respectively. The urinary bladder index was the highest in the group receiving CPX, in which it was over 3 times greater than in the control group, and the difference between that and the control was significant. Administration of carvedilol alone partly prevented the increase in bladder index (significant difference compared to the CPX and CON groups), but still it was almost 2.5 times greater than in the control group. The most pronounced prevention was found for groups receiving mesna. The bladder index increase in both cases was about 1.4 times greater than in the control group, but still it was significantly greater than in CON. However, the difference compared to the CPX group was also significant in the CPX-M and CPX-MC groups. The action of mesna was greater than carvedilol because significant differences were found between CPX-M vs. CPX-C and CPX-MC vs. CPX-C groups. There was no difference between the CPX-M and CPX-MC groups. In the CPX group, the kidney index was the highest and was significantly different from the CON group. Administration of carvedilol only partly prevented an increase in the kidney weight increase. The difference was still significant to the CON, but was also significant to the CPX group. Only groups receiving mesna (CPX-M and CPX-MC) significantly prevented the kidney index increase after CPX and the statistical differences between those and the CPX-receiving group were significant, but insignificant with the control group. The liver index was the highest in the CPX and CPX-C groups and was significantly different from the control group. The addition of mesna completely prevented the increase in the liver index (CPX-M vs. CON and CPX-MC vs. CON, *p* = NS in both cases). 

### 2.2. Serum Potassium and Creatinine Levels

**Figure 2:**
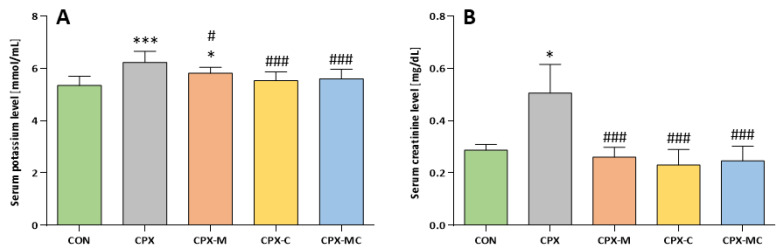
Serum potassium (**A**) and creatinine (**B**) levels on day 4 of the study. Data are presented as means ± SD. A detailed description of experimental groups is presented in Figure 1 and the Materials and Methods section. * *p* < 0.05, *** *p* < 0.001 vs. CON group; # *p* < 0.05, ### *p* < 0.001 vs. CPX group.

The highest serum potassium level on day 4 of the study was measured for the CPX group and was significantly different from CON. Administration of mesna alone only partly prevented the increase in serum potassium level (CPX-M vs. CON and CPX-M vs. CPX, *p* < 0.05 in both comparisons). Carvedilol given alone or in combination with mesna fully prevented the increase in the potassium level in serum and the differences between these two groups and the control group were insignificant, but there were significant differences with the CPX group. In the group receiving CPX, the serum creatinine level was the highest and was significantly different from the control group (1.76 times greater than in the CON). All studied substances fully prevented the serum creatinine level increase and the differences between these groups and CON were insignificant, but significantly different from the CPX group. The action of carvedilol, mesna, and the combination of the treatment was comparable, and no significant differences were noticed between these groups (*p* = NS in all comparisons). The mean values and SD for both parameters are presented in Figure 2A,B.

### 2.3. Hematuria, Urinary Bladder and Kidney Histology, Urinary Bladder and Kidney Scores

**Figure 3:**
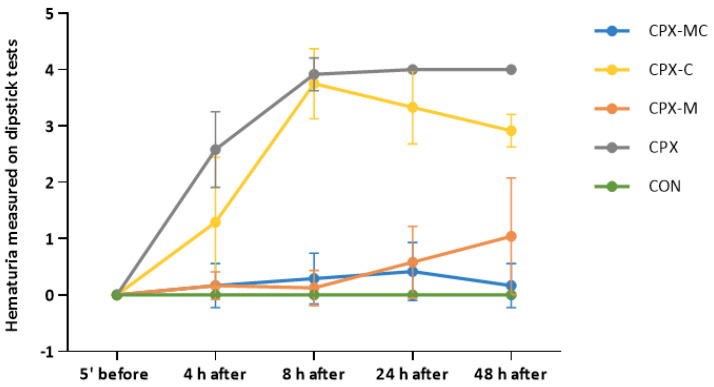
Hematuria measured semi-quantitatively on the dipstick test 5 min before, 4 h, 8 h, 24 h, and 48 h after injection of CPX in the studied groups or normal saline in the control group. Hematuria was evaluated from 0 to 4+ (0 means no hematuria detected and 4+ means macroscopically detectable hematuria). Data are presented as means ± SD. A detailed description of experimental groups is presented in Figure 1 and the Materials and Methods section, and detailed statistical comparisons are presented in Table 1.

**Figure 4:**
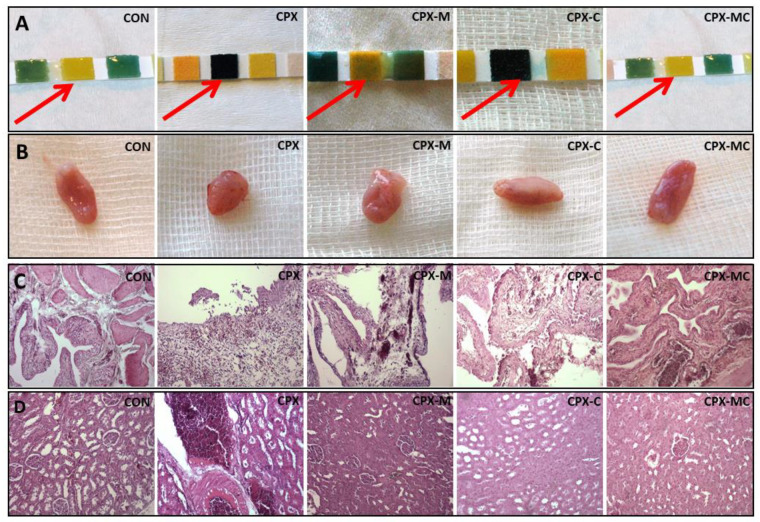
Representative results of dipstick tests for hematuria (**A**), macroscopic images of urinary bladders (**B**), microscopic images of urinary bladders (**C**), and microscopic images of kidneys (**D**). Histological images of bladders and kidneys (hematoxylin-eosin staining, 100× magnification). On dipstick tests (**A**) red arrows show the area of hematuria detection. In CON and CPX-MC groups no reaction is observed (yellow area), in CPX and CPX-C groups severe hematuria is noticed (the whole area is dark green), and in CPX-M group 1+ reaction is noticed (some green spots on the yellow area). Macroscopic images of urinary bladders (**B**) show no signs of edema in CON, CPX-M, and CPX-MC groups; urinary bladders from CPX and CPX-C groups are significantly swollen. Microscopic images of urinary bladders (**C**) reveal normal urothelium with normal lamina muscularis mucosae (CON group); inflammatory infiltrations, hemorrhages, and ulcerations (CPX group); hyperemia and thinning of the urothelium (CPX-M group); hyperemia, erosions, and thinning of the urothelium (CPX-C group); hyperemia with thinning of the urothelium (CPX-MC group). Microscopic images of the kidney (**D**) reveal hyperemia (CON group); large hemorrhages in the renal tissue (CPX group); swelling of tubules (CPX-M and CPX-MC groups), and atrophy of glomeruli (CPX-C group).

Before the intraperitoneal injection of CPX on the dipstick test, no hematuria was noticed in any of the studied groups. Significant hematuria was noticed in the CPX and CPX-C groups in comparison to the control group at 4 h, 8 h, 24 h, and 48 h after the injection of CPX. Intraperitoneal administration of mesna, in an equivalent dose to that of CPX, fully prevented the CPX-induced hematuria (significant difference compared to CPX, but insignificant to CON). The changes in hematuria in time in all experimental groups are presented in Figure 3. Representative images of dipstick tests of hematuria and macroscopic changes in urinary bladders (just after resection) are presented in Figure 4A,B, respectively.

**Figure 5:**
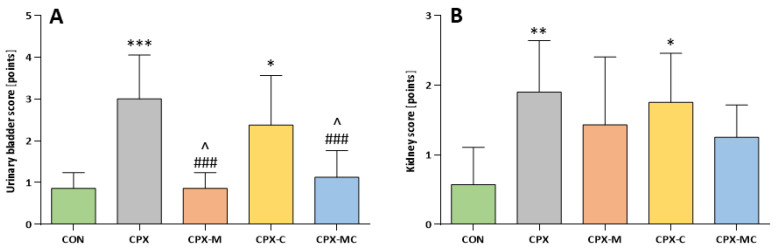
Urinary bladder score (**A**) and kidney score (**B**) based on histological injuries evaluation. The scoring system is presented in the Materials and Methods section. A detailed description of experimental groups is presented in Figure 1 and the Materials and Methods section. * *p* < 0.05, ** *p* < 0.01, *** *p* < 0.001 vs. CON group; ### *p* < 0.001 vs. CPX group; ^ *p* < 0.05.

In the microscopic evaluation of urinary bladders, in almost all studied specimens focal or diffuse urothelium reduction were found; therefore, these changes were not involved in the scoring system as they were nonspecific to any of the performed experimental procedure. The most pronounced changes in a form of inflammatory infiltrations, edema, hemorrhage, or erosions were found in the group receiving only CPX. Additionally, ulceration of urothelium was found in one case. Considering the scoring system of histological changes in urinary bladders, the highest score was calculated for the CPX group and was significantly different from the control group. Carvedilol did not prevent histological injury in urinary bladders and the score value in the CPX-C group was not significantly different from the CPX group, but was significantly different from the CON group. Administration of mesna alone or in combination with carvedilol fully prevented the histological injury and reversed the CPX-induced changes. A significant difference between groups receiving mesna was found in comparison to the CPX-C group. The representative images from urinary bladder histological evaluation are presented in Figure 4C. The mean values with the corresponding SD for urinary bladder score are presented in Figure 5A.

The histological changes in the kidney were less specific and in almost all studied specimens parenchymatous offuscation and hyperemia were described, and similarly to the bladder scoring system, these two parameters are not involved as they are not dependent on experimental procedures. The most pronounced pathologies were described in the CPX-receiving group, where dilated urinary spaces, interstitial hemorrhages, and glomerular atrophy were found in almost all examined samples. Considering the scoring system CPX caused significant injury when compared to the control group (*p* < 0.01 vs. CON). Administration of mesna (alone or with carvedilol) partly reversed the pathological changes expressed in scoring (in both cases the differences between mesna-receiving groups to CON and CPX groups were insignificant). Carvedilol alone did not exert any effect on CPX-induced damages (*p* < 0.05 vs. CON, and *p* = NS vs. CPX group). However, these two groups (CPX-C and CPX) differ in such a way that in the group receiving CPX, interstitial hemorrhages were found in half of the specimens, whereas in the CPX-C group, interstitial hemorrhage was noticed only in one case. From all groups receiving studied substances, the least pronounced injuries were described in the group receiving both mesna and carvedilol, where no cases of interstitial hemorrhage were found. The representative images from kidney histological evaluation are presented in Figure 4D. The mean values with the corresponding SD for kidney score are presented in Figure 5B.

### 2.4. Oxidative Stress Parameters in Urinary Bladder, Kidney and Liver and Kidney IL-1β Level

**Figure 6:**
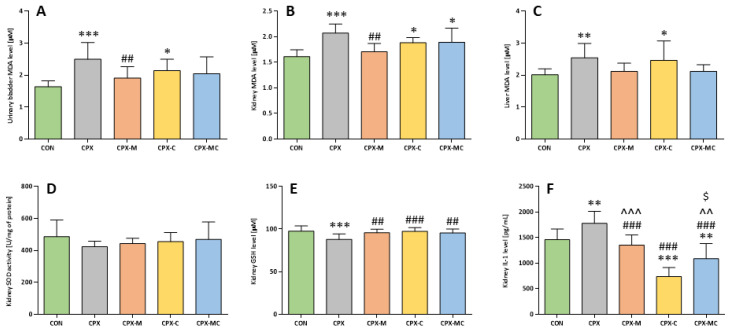
Malondialdehyde (MDA) levels in urinary bladder (**A**), kidney (**B**) and liver (**C**) homogenates, superoxide dismutase (SOD) activity (**D**), glutathione (GSH) level (**E**), and interleukin 1β (IL-1β) level (**F**) in kidney homogenates. Data are presented as means ± SD. A detailed description of experimental groups is presented in Figure 1 and in the Materials and Methods section. * *p* < 0.05, ** *p* < 0.01, *** *p* < 0.001 vs. CON group; ## *p* < 0.01, ### *p* < 0.001 vs. CPX group; ^^ *p* < 0.01, ^^^ *p* < 0.001 vs. CPX-C group and ^$^ *p* < 0.05 vs. CPX-M.

After the single intraperitoneal injection of CPX at a dose of 200 mg/kg, the malondialdehyde (MDA) level was significantly increased in all studied tissues (urinary bladder, kidney, and liver). Administration of mesna alone (group CPX-M) completely prevented the CPX-induced increase in MDA level in urinary bladder and kidney (*p* = NS vs. CON, *p* < 0.001 vs. CPX, in both tissues) and partly reversed the CPX action in the liver (*p* = NS vs. CON and CPX). In none of the examined tissues was administration of carvedilol able to reverse the increased level of MDA caused by CPX (significant difference compared to the control group). The drug combination (group CPX-MC) only prevented the CPX-induced changes in MDA level in the urinary bladder and liver (*p* = NS vs. CON and CPX) but exerted no effect in the kidney (*p* < 0.01 vs. CON, *p* = NS vs. CPX). The mean values with the corresponding SD for urinary bladder, kidney, and liver MDA levels are presented in Figure 6A–C, respectively.

CPX caused a significant decrease in glutathione (GSH) concentration in kidney homogenates (*p* < 0.001 vs. CON) and both studied substances (mesna and carvedilol, alone or in combination) reversed this action, increasing the GSH concentrations (CPX-M vs. CPX and CPX-MC vs. CPX, *p* < 0.001 in both cases, and CPX-C vs. CPX, *p* < 0.01). It was also noticed that injection of CPX decreased superoxide dismutase (SOD) activity and the value was the lowest in this group from all experimental groups, but the difference between that and the control group was not significant. In the kidney, the tendency to increase SOD activity in comparison to the CPX group was observed in the case of all groups receiving studied compounds, but there were only insignificant differences regarding the CON and the CPX group. The mean values with corresponding SD for kidney GSH levels and SOD activity are presented in Figure 6D,E, respectively.

In the kidney, CPX significantly increased the level of IL-1β in comparison to the control group. Administration of mesna alone failed to reverse this action, but administration of carvedilol not only prevented a CPX-induced increase in IL-1β level (CPX-C vs. CPX and CPX-MC vs. CPX, *p* < 0.001), but additionally, significantly decreased the level of this cytokine in kidney homogenates in comparison to the control group, because the IL-1β levels were lowest in groups receiving carvedilol, especially alone (CPX-C vs. CON, *p* < 0.001 and CPX-MC vs. CON, *p* < 0.01). The differences between the CPX-C group and both groups receiving mesna (CPX-M and CPX-MC) were also significant. The mean values with corresponding SD for kidney IL-1β levels are presented in Figure 6F.

### 2.5. Asymmetric Dimethylarginine (ADMA) and Dimethylarginine Dimethylaminotransferase (DDAH) Assessment

**Figure 7:**
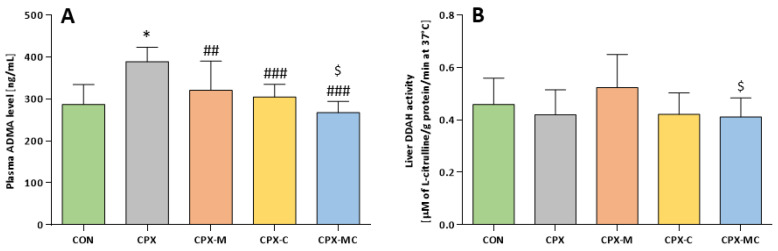
Plasma ADMA level (**A**) and DDAH activity (**B**) in liver homogenates. Data are presented as means ± SD. A detailed description of experimental groups is presented in Figure 1 and in the Materials and Methods section. * *p* < 0.05 vs. CON; ## *p* < 0.01, ### *p* < 0.001 vs. CPX group; ^$^ *p* < 0.05 vs. CPX-M.

Administration of CPX significantly increases the plasma ADMA level. All studied substances (mesna and carvedilol given alone or together) completely prevented the CPX-induced ADMA increase (*p* = NS vs. CON in all treated groups and significant differences between the others and the CPX group). Additionally, the ADMA level is the lowest in the CPX-MC group, significantly lower not only compared to the CPX group but also compared to the CPX-M group (CPX-MC vs. CPX-M, *p* < 0.05). No statistically significant differences in liver DDAH activity in comparison to the control group were noticed. However, the highest liver DDAH activity was measured in the CPX-M group, but it was still insignificant. Serum ADMA level and liver DDAH activity are presented in Figure 7A,B, respectively.

## 3. Discussion

**Figure 8:**
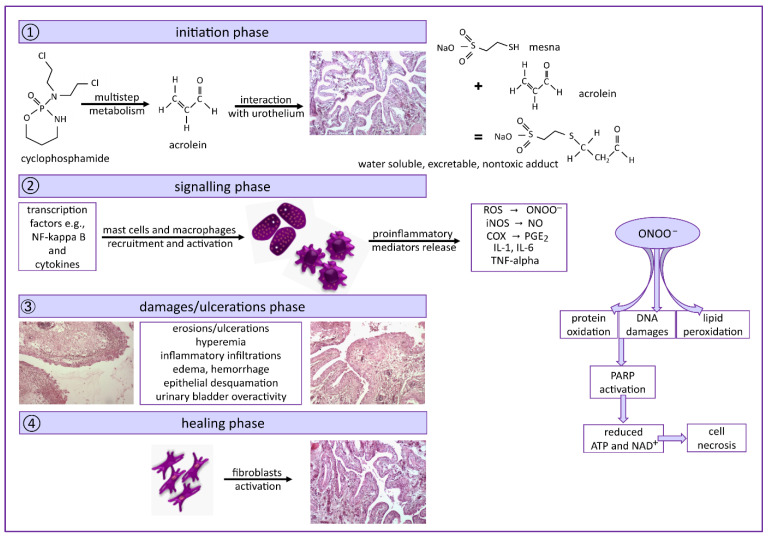
The proposed mechanism of urinary bladder toxicity induced by cyclophosphamide, based on [7,31,32,33]. ROS—reactive oxygen species, ONOO^–^—peroxynitrite, iNOS—inducible nitric oxide synthase, NO—nitric oxide, COX—cyclooxygenase, PGE_2_—prostaglandin E_2_, IL-1—interleukin 1, IL-6—interleukin 6, TNF-α—tumor necrosis factor-alpha, NF-κB—nuclear factor kappa B, PARP—poly (ADP-ribose) polymerase, ATP—adenosine triphosphate, NAD^+^—an oxidized form of nicotinamide adenine dinucleotide. Images magnification 100×.

Based on many earlier experimental data, Ribeiro et al. [7] proposed a four-stage model of hemorrhagic cystitis induced by cyclophosphamide, similar to the model of mucositis (Figure 8). It involves the interaction of acrolein (the toxic metabolite of CPX) accumulated in the bladder with urothelium and urothelial damage, followed by up-regulation of a variety of transcription factors and proinflammatory cytokines (mainly TNF-α and IL-1β) release from recruited macrophages. Large amounts of cytokines, ROS generation, and overexpression of iNOS as well as COX-2 lead to the overproduction of nitric oxide (NO) and peroxynitrite. In the phase of clinical symptoms, erosions and ulcerations in the urothelium are present, which results in pain and bladder dysfunction. The last phase is the healing phase, involving fibroblasts and locally released growth factors [7,31,32,33]. 

Acrolein is not solely the product of cyclophosphamide liver metabolism, but is also formed endogenously during oxidative stress-mediated lipid peroxidation, myeloperoxidase-mediated oxidation of amino acids, or may be of the environmental origin as a pollutant in tobacco smoke or from other sources [34,35,36]. Acrolein is a very reactive molecule physiologically rapidly neutralized. Conjugation with glutathione is the most important pathway, counting for about 60–70% of acrolein metabolism [37]. It is postulated that acrolein may be involved in endothelial dysfunction [34,38], may interfere with NO production, and may increase oxidative stress [39]. Another aspect of acrolein toxicity is found in the reproductive system, where acrolein induces apoptosis in male germ cells, Sertoli cells, or Leydig cells [35,36,40]. 

In clinical practice, mesna and/or hydration with or without diuretics are commonly used to prevent dose-dependent cyclophosphamide-induced urinary toxicity such as micro- and macrohematuria or hemorrhagic cystitis [5,14,41], which may be observed even in up to 75% of patients receiving very high intravenous doses of cyclophosphamide [12,14,42]. The recommended total dose of mesna in humans is, in most cases, 60% (*w*/*w*) of the CPX dose given in three divided doses (15–30 min before, 4 h and 8 h after the oxazaphosphorine), but may be increased up to 160% of the CPX dose in four divided doses or even up to 320% of the daily post-transplant cyclophosphamide dose [5,41,43]. However, the efficacy of mesna use is currently being discussed, especially considering the CPX-induced urinary complications in the treatment of chronic diseases [15,16]. Additionally, mesna may cause some skin and systemic hypersensitivity-like-reactions, but it is sometimes difficult to distinguish these from the adverse effects of the basic anticancer protocol [41,44]. Therefore, there is still a great need to search for new compounds which may be active in alleviating CPX-induced cystitis. The first idea was to search among other drugs with thiol groups, such as amifostine or N-acetylcysteine. Amifostine is registered for the prevention of cisplatin-induced nephrotoxicity [45], and some studies suggest that it may be also effective in CPX-induced bladder toxicity [42]. N-acetylcysteine is used in the treatment of paracetamol overdose [46], but the newest study of Dobrek et al. [11,47] revealed protective action of N-acetylcysteine in a model of acute and chronic CPX-induced bladder and kidney toxicity. N-acetylcysteine was found to protect not only against CPX-induced urinary toxicity but was effective in CPX-induced cardiotoxicity [48]. 

Due to the very complex mechanism of CPX-induced urinary toxicity, research focuses on substances inhibiting, e.g., the inflammation cascade or overactivation of oxidative stress. Introducing new natural or synthetic compounds to clinical practice takes a long time of preclinical and clinical tests. Searching for new applications among already registered drugs with known toxicity and pharmacokinetic is a promising way to shorten this time [49]. In experimental studies, such attempts were made, for example, with ambroxol [50], pantoprazole [51], or ketamine [52]. Carvedilol has been known since the 1980s [53] and is currently used in the treatment of essential hypertension, chronic stable angina pectoris, or heart failure [54]. For the last almost 40 years in the history of carvedilol clinical administration, many new properties of this drug, other than only beta non-selective and selective alpha-1 adrenergic blockade were described [19,54]. These features open new possibilities for the use of carvedilol in indications other than cardiovascular. Carvedilol is a potent antioxidant, metabolized to metabolites with 30–100 times greater [19,30] antioxidant activity than a parent compound. It has both ROS-scavenging properties and ROS-suppressing effects. The carbazole group in the carvedilol structure is responsible for antioxidant activity, and high lipophilicity enables the access of the drug to the intramembrane sites which are targets for lipid peroxidation [55]. Carvedilol is unique, having anti-inflammatory, antiapoptotic, antifibrotic or anticancer activity [17,30,49,56,57]. Well described is carvedilol prevention from heart injury caused by anthracyclines [20,56]. Many papers describe the alleviation by carvedilol kidney injury caused by ischemia/reperfusion (I/R) [58,59], cisplatin [26,60], or azithromycin [23]. Some works outline the protective action of carvedilol in other organs, such as the pancreas of diabetic animals [61,62]. The postulated mechanisms of carvedilol action are different and cover not only the antioxidant activity but also decrease in TNF-α and other proinflammatory or fibrogenic cytokines (e.g., IL-1β, IL-6, IL-18, TGF-β1), increase in anti-inflammatory cytokines (e.g., IL-10), decrease expression of MMP-2 and MMP-9, COX-2, iNOS or caspase-3 [23,25,29,57,62,63].

In the present study, we have chosen a low dose of carvedilol (2 mg/kg) given once daily by gastric tube for 3 days. The low dose was selected based on the earlier published papers confirming that an intraperitoneal dose of 2 mg/kg given in 12 h intervals is effective in the prevention of I/R kidney injury in rats where carvedilol restored antioxidants levels (SOD, GSH, catalase) and decreased MDA level in kidney tissue altered by I/R procedure. Moreover, doses from 1 to 3 mg/kg exerted the least pronounced hemodynamic effect, whereas higher doses did not give additional protection in the I/R model but produced a decrease in systolic blood pressure and heart rate [64]. The same oral dose of carvedilol was examined by Hayashi et al. [58,59] in a similar model of kidney I/R with benefits for serum creatinine and kidney SOD activity. What is more, Watanabe et al. [65] described better protection of 2 mg/kg dose of carvedilol over 20 mg/kg dose in a rat model of dilated cardiomyopathy. These findings prompted us to choose just this dose, administered intragastrically for three consecutive days, in the model of CPX-induced urinary toxicity. The efficacy of 2 mg/kg dose of carvedilol was also confirmed in the model of acute septic kidney injury [66] where, among others, a reduction in kidney MDA and an increase in GSH levels were found, as well as improvement in serum creatinine without an impact on baseline blood pressure.

In our study, the model of a single intraperitoneal injection of 200 mg/kg of CPX was used to induce hemorrhagic cystitis and kidney injury as it is described in some other papers [42,47,48,67,68,69]. In our experiment, CPX caused a significant decrease in total body weight with a significant increase in urinary bladder index. Ozatica et al. [68] had similar results after the same dose of CPX. However, in some other papers only increased bladder index was noticed without decrease in total body weight of rats after injection of 200 mg/kg of CPX [69]. These differences may be due to the fact that Elrashidy et al. [69] sacrificed animals 24 h after CPX administration; in our work it was 48 h, whereas in the work of Ozatic et al., it was 7 days. Nevertheless, a significant decrease in total body weight after CPX observed in our experiment reveals important general toxicity of CPX. Increased bladder index indicates tissue swelling, which was also found macroscopically. Murali et al. [70] noticed a decrease in body weight and increase in bladder weight after 4, 24, and 48 h after injection of CPX in a mice model of urotoxicity, and this action was also significantly reversed by mesna and data were consistent with the macroscopic evaluation of bladders. The addition of mesna in our experiment partly prevented CPX-induced changes in both parameters. This is consistent with other published data, in which mesna administration reversed the CPX-induced bladder edema [69,71]. Furthermore, in our study, mesna alone or combined with carvedilol completely reversed the hematuria measured semi-quantitatively on a dipstick test, but carvedilol alone has no hematuria-preventive action. Administration of carvedilol partly reversed both decrease in body weight and bladder index increase. We did not observe any synergism of mesna and carvedilol, but the mean bladder index was the lowest in both groups receiving mesna and significantly lower not only in the CPX group, but also in the CPX-C group. As expected, CPX caused a significant increase in bladder MDA level, which is consistent with other studies [9,51]. What is surprising is that, despite many reports about the antioxidant activity of carvedilol in, e.g., doxorubicin-induced cardiotoxicity [25] or in mice model of diabetes [62], we did not observe a decrease in bladder MDA concentration. The differences may result from given doses; 2 mg/kg for 3 days in our model and much greater doses of 15–20 mg/kg for 14 days in a study by Amirshahrokhi et al. and even 30 mg/kg for 6 weeks in a study by Alanazi et al. However, as mentioned earlier, some authors noted the MDA decrease after only two intraperitoneal doses of 2 mg/kg of carvedilol [64]. The model used in our experiment was most of all designed to evoke acute cystitis, which was confirmed in histological evaluation of bladders. In the CPX group edema, inflammatory infiltrations, erosions, ulcerations, and hemorrhage were noticed. Similarly, mucosal erosions, ulceration, submucosal edema, hemorrhagic foci, and leukocyte infiltration were described by other authors in the same model CPX injection [67,68,69] or our previous paper after the 10-day-long CPX administration at a dose of 15 mg/kg daily [72]. Only treatment with mesna completely reversed the histological injury caused by CPX and this action was not only significant in comparison to the CPX, but also to the CPX-C group. This was confirmed by a significant decrease in hematuria and histological bladder score. Mesna reversed the CPX-evoked damages in many other models of bladder injury [51,69]. Carvedilol given alone did not exert any protection from CPX-induced cystitis either in bladder score and histologically described changes or in reducing the hematuria. It is difficult to compare our results to other authors’ findings, since in the available for us databases we did not find any paper describing the action of beta adrenolytic drugs on urothelium affected by cyclophosphamide.

CPX, in contrast to ifosfamide, is not uptaken by kidney proximal tubules and the nephrotoxic action of CPX is rather due to the generation of ROS and peroxynitrite, activation of inflammatory pathways, cell membrane disruption, mitochondrial dysfunction and DNA or protein adducts formation by acrolein [73]. In the study we performed, cyclophosphamide caused a significant increase in kidney index which indicates that the edema of the renal tissue, and similar changes were described by Dobrek et al. [11] in a rat model or by Jiang et al. [74] in a mice model of chronic CPX administration. In the CPX group, we noticed a significant increase in serum potassium and creatinine levels which was reported by many other authors after CPX injection to rats [10,75,76] or mice [77,78,79] in both acute and chronic models of CPX administration. Mesna alone only partly decreased the potassium level, but carvedilol alone or in combination with mesna completely reversed the CPX-induced alteration in serum potassium. In the case of creatinine, both mesna and carvedilol, given alone or in combination, prevented a CPX-induced increase. Carvedilol was shown to prevent an increase in creatinine level in other experimental models, e.g., I/R kidney damage [58,59] or acute septic injury [66], and our study confirmed this preventive activity in the model of acute cyclophosphamide administration. The action of mesna is not surprising, and a nephroprotective effect was described with similarly acting N-acetylcysteine [11,47]. Cyclophosphamide caused alterations in oxidative stress and inflammatory parameters evaluated in our study. We found a significant increase in kidney IL-1β level, MDA concentration with a significant decrease in kidney GSH level and an insignificant decrease in kidney SOD activity. Mesna was potent in reversing all these abnormalities in kidney tissue and very similar results were obtained by Elrashidy et al. [69] in the urinary bladder, by Ghareeb et al. [79] in the kidney, or by Hagar et al. [80] in the pancreas. The antioxidant properties of mesna are comparable to ascorbic acid, a very well-known antioxidant, and were also confirmed in in vitro studies [81]. Increasing GSH concentration is of great value; as mentioned earlier, GSH is the most important pathway to detoxifying acrolein [37]. What is surprising is that despite the well documented antioxidant action of carvedilol in various models of tissue injury [25,57,62,64], in our study we did not notice the impact of carvedilol on CPX-evoked increase in kidney MDA level; we found only a significant restoration of renal GSH. We cannot exclude that a longer treatment with a low dose of carvedilol is necessary for the full antioxidant effect to be revealed. Interleukin 1β is an important proinflammatory cytokine and its level is increased after CPX administration in serum [75,76], urinary bladder [69,82], kidney [75,83], and many other tissues [84]. Carvedilol is potent in decreasing IL-1β concentration or mRNA expression in the model of brain I/R injury [85], experimental myocardial infarction [63], drug-induced cardiotoxicity [23,57], diabetes [62], or even in the rat model of periodontitis [22]. Despite so many published papers, we did not find anything about the action of carvedilol on CPX-induced increase in kidney IL-1β concentration or expression. In one very recently published study by Wanas et al. [83], nebivolol, another third-generation beta adrenolytic, decreased the IL-1β level in rat kidneys. Renal toxicity of cyclophosphamide was confirmed by us in histological evaluation. In the CPX group, we found interstitial hemorrhages, dilated urinary spaces, and glomerular atrophy to be the most pronounced pathology. Additionally, in groups receiving CPX, different levels of the swelling tubules were noticed. Similarly, other authors described swelling of proximal convoluted tubules, inflammatory cells infiltration, tubular and glomeruli atrophy, tubular degeneration, and dilatation in Bowman’s capsules [76,77,78,79,83]. In contrast to our study, Dobrek et al. [47] did not reveal any significant pathology in the rat kidneys after the same intraperitoneal dose of CPX. The main difference was time of observation after CPX dose; 24 h in the previously mentioned study and 48 h in our experiment. In humans, the plasma half-life of CPX is from 4 to 8 h, which means that up to 40 h may be needed to eliminate a single dose. The plasma half-life of the active metabolites of CPX is not defined [2]. In our study, the exposure of kidneys to CPX was longer than in a study of Dobrek et al. [47]. As expected, mesna significantly reversed the CPX-induced changes, which was confirmed in the kidney score. In contrast to nebivolol [83], carvedilol did not attenuate significantly histological changes caused by CPX in kidneys, except for decreasing incidents of interstitial hemorrhages. It seems, however, that the presence of kidney pathologies does not 100% correlate with kidney function, as carvedilol alleviates CPX-induced changes in serum potassium and creatinine concentrations, which can be interpreted as improvement of kidney function. Dobrek et al. [11] noted that N-acetylcysteine improved CPX-induced alterations in kidney function, although it did not significantly improve histopathological abnormalities. Inflammatory cells infiltration or hemorrhages in tissues after administration of CPX are not only characteristic for urinary bladder or kidney but are also present in other tissues, e.g., in the heart [84]. 

Liver toxicity was not the primary goal of our study, but we found significantly increased liver index in the CPX group, which was reversed only by mesna, but not by carvedilol alone. Cyclophosphamide may cause abnormal hepatic function and very rarely hepatitis and hepatomegaly [2]. A lot of research focused on finding natural, endogenous, or synthetic compounds that may decrease CPX-induced liver toxicity interfering with different mechanisms of injury [86,87,88]. We revealed only the protective action of mesna in reversing CPX-induced liver index increase and on liver MDA concentration. Carvedilol failed to prevent changes caused by CPX in the liver and in the case of MDA level, the explanation may be the same as in the case of bladder MDA level, which was discussed earlier. The protective action of mesna on CPX-induced increase in the liver index or MDA level may be explained by the aforementioned acrolein-binding properties. Acrolein was found to exert toxic action on the liver, affecting, among others, mitochondria of hepatocytes [89]. Al-Jawad et al. [90] confirmed the hepatoprotective effect of N-acetylcysteine in a carbon tetrachloride (CCl_4_) model of liver injury. Despite some reports about the hepatoprotection of carvedilol in other animal models of liver damage, such as CCl_4_-induced fibrosis [91] or I/R injury [92], our preliminary data did not confirm it, but a broader panel of studied parameters is necessary in order to make conclusions.

The protein arginine methyltransferase (PRMT) enzymes catalyze the formation of ADMA [93]. ADMA is an inhibitor of all nitric oxide synthases isoforms, including eNOS resulting in decreased NO production [94]. Increased level of ADMA is considered as the determinant of endothelial dysfunction and a risk factor of cardiovascular disorders [95]. Additionally, ADMA increases the NFκB expression causing the enhanced synthesis of proinflammatory cytokines [96]. Generated ADMA is metabolized predominantly (in about 80%) by DDAH to citrulline and dimethylamine or excreted with the urine (in less than 20%) [94,96]. DDAH is expressed in kidneys and many other tissues, e.g., the liver [97,98]. Still, little is known about the impact of cyclophosphamide administration on plasma ADMA level. In our previous work, we found that intragastrical administration of 15 mg/kg daily dose of CPX for 10 days caused significant a decrease in serum ADMA level with a significant increase in liver DDAH activity [99]. This was, however, a very different model of CPX administration than we used in the current study, in which a single dose of 200 mg/kg of CPX was administered intraperitoneally. In this study, we noticed a significant increase in plasma ADMA level without impact on DDAH liver activity. Similar results of plasma ADMA concentration were reported by Mansour et al. [48] in rats injected intraperitoneally with 200 mg/kg CPX dose. Because ADMA is only in a small amount excreted with kidneys, DDAH metabolism is one of the most important ways to decrease ADMA plasma levels [94,96]. In our study, we did not notice a significant decrease in DDAH activity in the liver; however, it was the lowest in the CPX-receiving group out of all the studied groups. Probably the reason for increased ADMA level in the CPX group is something other than inhibition of liver DDAH activity. DDAH is present in kidneys in the renal proximal tubules [96,98,100], so unaffected kidney function is important for ADMA clearance in both, metabolism, and excretion [100]. It was shown in some clinical studies that impaired kidney function is associated with increased plasma ADMA levels [101,102,103]. It cannot be excluded that an increase in ADMA is caused by decreased kidney DDAH activity, which was not evaluated in the present study. However, Betz et al. [104] did not find the altered renal expression of DDAH or PRMTs in the acute rat model of ischemia/reperfusion kidney injury, but it was different from our model of injury. On the other hand, Wang et al. found decreased DDAH2 expression with increased PRMT1 expression in kidneys of Zucker diabetic fatty rats [105] or two-kidney, one-clip (2K1C) rat model of kidney injury [106] accompanied with increased ADMA plasma and kidney levels. We cannot completely rule out that the acutely impaired kidney function may, at least partly, be responsible for the effect observed in our study. This requires further confirmation with a renal expression of PRMT and/or DDAH. Data concerning the action of carvedilol on plasma ADMA level are still rather scant and contradictory. Alfieri et al. [107] noticed the significant difference in plasma ADMA concentration after carvedilol in patients with heart failure, but only in the group responding to carvedilol treatment. On the contrary, in a group of patients with interdialytic hypertension, carvedilol was found to improve endothelial cell function but did not change plasma ADMA levels [108]. Data from animal experimental models on the impact of carvedilol on ADMA levels are even more limited. More information is found for another beta adrenolytic with vasodilating properties: nebivolol. In the 2K1C rat model of kidney injury, nebivolol reversed increased plasma and kidney ADMA levels, additionally increasing DDAH2 and decreasing PRMT1 kidney expression [106]. Very similar results were obtained in the kidney injury in Zucker diabetic fatty rats [105]. Nebivolol also decreased serum ADMA levels in the cyclosporine-injured kidneys [109]. In our study, intragastrical administration of carvedilol completely reversed the CPX-induced increase in plasma ADMA levels, without changing the activity of liver DDAH. Nebivolol and carvedilol have some common properties, e.g., both dilate blood vessels, release NO and have antioxidant effects [110], which may explain the action of carvedilol on ADMA plasma concentration observed in the current study. Additionally, in our experiment, carvedilol improved kidney function, which was reflected in the prevention of CPX-induced increase in serum potassium and creatine concentrations and the exertion of some antioxidant and anti-inflammatory activity, preserving kidney GSH levels and decreasing IL-1β concentration in kidney homogenates. It is certain that further studies on the exact mechanism and kidney DDAH/PRMT expression or intrarenal ADMA level in CPX-induced nephrotoxicity are required. To the best of our knowledge, nothing was published about the effect of mesna on the ADMA/DDAH pathway. Some experimental data highlight the protective action of N-acetylcysteine [48]. The activity of N-acetylcysteine in reducing ADMA serum and kidney levels and in restoring kidney DDAH levels/activity was found in a mice model of renal I/R injury [111]. Administration of N-acetylcysteine to the hemodialyzed patients also decreased serum ADMA [112]. However, the closest to our research model is the model of CPX-induced cardiotoxicity in rats used by Mansour et al., in which administration of 200 mg/kg of N-acetylcysteine completely prevented the increase in serum ADMA caused by a single injection of CPX [48]. Mesna, as with N-acetylcysteine, contains -SH groups and is an antioxidant [81]. In our experiment, administration of 200 mg/kg of mesna in divided doses reduced the CPX-induced plasma ADMA level. When mesna was given in combination with carvedilol, the mean value of ADMA in the CPX-MC group was the lowest out of all the studied groups and was significantly lower than in the CPX-M group. Mesna did not significantly influence the liver DDAH activity, but, as it was stated earlier, to draw more accurate conclusions, the kidney expression of DDAH and PRMT in the CPX-induced model of tissue injury should be further investigated.

Carvedilol exerts many properties that can be important in protecting against CPX-induced urotoxicity. Despite the complex mechanism, we did not find significant protection against CPX-evoked cystitis or potentiation of mesna action. It cannot be excluded that direct neutralization of acrolein than antioxidant activity of carvedilol is more important for the prevention of cystitis. Despite a detailed review of the literature available to us, we did not find any paper describing the action of carvedilol in CPX-induced cystitis or nephrotoxicity, so it is difficult to compare our results with other studies. Some reports confirm that carvedilol prevents toxicity induced by other anticancer agents, especially in the doxorubicin- or daunorubicin-induced cardiotoxicity [25,56], cisplatin-induced nephrotoxicity [26,60], testicular damage [27], or aspirin-induced gastrotoxicity [29]. In this context, our results seem to be novel, but a little bit surprising and contrary to expectation, and more detailed studies should be provided, especially in the field of carvedilol potential as a nephroprotective agent.

## 4. Materials and Methods

### 4.1. Chemicals

Cyclophosphamide *subst*. (Sigma, Steinheim, Germany), mesna *subst*. (Sigma, Steinheim, Germany), carvedilol *subst*. (Sigma, Steinheim, Germany), 0.9% NaCl solution, ampules 10 mL (Polpharma S.A. Starogard Gdański, Poland), pentobarbital sodium 133.3 mg/mL + pentobarbital 26.7 mg/mL, bottles 100 mL (Morbital^®^, Biowet, Puławy, Poland).

### 4.2. Animals

Male Wistar rats with an average weight of 315.67 g ± 17.98 g were purchased from the Animal Research Center at Wroclaw Medical University (Wrocław, Poland) and after 2 weeks of adaptation period, they were randomly divided into five experimental groups (12 animals/per group). Rats were housed two per cage in standard laboratory conditions of 21–23 °C, 12 h:12 h light:dark cycle, free access to standard rodent feed (Agropol, Motycz, Poland), and water ad libitum. The experiment was approved by the Local Ethics Committee for Animal Experiments in Wrocław (No. 13/2012). 

### 4.3. Experiment Design

**Figure 9:**
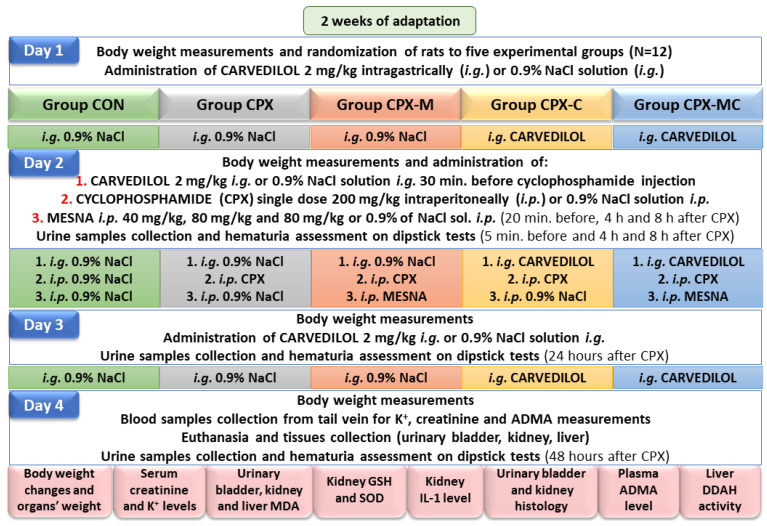
The general organization of the experiment.

Hemorrhagic cystitis in rats was induced by a single intraperitoneal (*i.p.*) injection of 200 mg/kg of CPX. Mesna, as a reference treatment, was given *i.p.* 20 min before, 4 h and 8 h after CPX administration at a total dose of 200 mg/kg divided in 20%, 40%, and 40% doses, respectively, which was equal to 40 mg/kg, 80 mg/kg, and 80 mg/kg for separate injections. CPX and mesna were dissolved in normal saline and administered in a 5 mL/kg volume. Normal saline solution given intraperitoneally in the same volume and schedule served as a control. Carvedilol was given intragastrically (*i.g.*) by gastric tube (FST, Foster City, CA, USA) once daily at a dose of 2 mg/kg, dissolved in 4 mL/kg normal saline solution, a day before, on the day, and a day after CPX administration. Animals in the control group were given normal saline solution *i.g.* at the same volume and schedule. Animals were sacrificed by cervical dislocation in barbiturate anesthesia 48 h after the CPX administration. Before the CPX injection and after 4 h, 8 h, 24 h, and 48 h, urine samples were taken for assessment of hematuria. Blood samples from the tail vein were collected 24 h before CPX injection and 48 h after. Urinary bladders, kidneys, and livers were separated, weighed, and collected for further histological evaluation and preparation of tissue homogenates. The general organization of the experiment and the nomenclature of the experimental groups is summarized in Figure 9.

### 4.4. Body Weight and Organ Weight

Body weight (BW) was checked and recorded (in grams) once daily in the morning from day 1 to day 4 of the study. After the animals were sacrificed, the urinary bladders, kidneys, and livers were collected and weighed. The urinary bladder, kidney, and liver weights were expressed as the percentage of the total body weight (urinary bladder index, kidney index, and liver index) from day 4 and calculated according to the formula:

$$Tissue\;index=\frac{organ\;weight\;\left(g\right)}{total\;body\;weight\;\left(g\right)}\;\times \;100\%$$

### 4.5. Serum Potassium and Creatinine Levels

Forty-eight hours after the CPX administration, blood samples were collected from the tail vein for serum potassium and creatinine measurements. The measurements in serum were made after centrifugation in the certified commercial laboratory just after the samples were collected.

### 4.6. Hematuria

Urine samples for hematuria evaluation were collected directly on the Siemens Multistix 10SG test (Siemens Healthcare, Erlanger, Germany) by a gentle massage of suprapubic area 5 min before CPX or normal saline injection, and 4 h, 8 h, 24 h, and 48 h after CPX or normal saline injection. The magnitude of hematuria was assessed semi-quantitatively (from 1+ to 4+) using the scale on the dipstick, modified in this way, so that 4+ were given when macroscopic hematuria was noticed.

### 4.7. Oxidative Stress Parameters in Urinary Bladder, Kidney and Liver and Kidney IL-1β Level

Part of the urinary bladder, kidney and liver tissues were homogenized on ice, with lysis buffer (140 mM NaCl, 10 mM EDTA, 10% glycerol, 1% NP40, 20 mM Tris base, pH 7.5) and thereafter centrifuged at 14,000 rpm for 25 min at 4 °C using homogenizer Pro250 (Pro Scientific Inc., Oxford, CT, USA). MDA, GSH, SOD, and IL-1β were assayed in supernatants. MDA concentrations were assessed in the urinary bladder, kidney, and liver tissues, whereas GSH concentrations and SOD activity were determined in kidney tissue using the kit and following the instructions of the manufacturers on the MARCEL S350 PRO spectrophotometer (Marcel S.A., Sp. z o.o., Zielonka, Poland). MDA and GSH were assayed using BIOXYTECH-MDA-586 kit and BIOXYTECH GSH-400, respectively (OxisResearch, Portland, OR, USA), and their concentrations were expressed in μM. The activity of SOD was evaluated using the Ransod kit (Randox Laboratories, Crumlin, UK) following the manufacturer’s instruction, and activity was expressed as U/mg of protein. Total protein concentration in supernatants was measured in a certified laboratory using the Dimension RxL-Max (Siemens Healthineers Nederland B.V., Den Haag). In brief, in an alkaline solution, cuprum interacts with peptide bond in protein; the amount of measured Cu(II) complex is proportional to protein concentration.

Interleukin 1β in kidney tissue was assessed by Rat IL-1β ELISA kit (DIACLONE SAS, Besançon, France) according to the manufacturer’s instructions on Epoch ELISA Reader (BioTek Instruments, Winooski, VT, USA) and the results were expressed in pg/mL.

### 4.8. ADMA and DDAH Assessment

On the last day of the study, the blood samples from the tail vein were collected and plasma ADMA level was evaluated using E91301Ge ELISA Kit (USCN, Life Science Inc., Houston, TX, USA) according to the manufacturer’s instruction and the results were expressed in ng/mL. Plasma ADMA concentrations were assessed on Epoch ELISA Reader (BioTek Instruments, Winooski, VT, USA).

DDAH activity was determined in liver tissue homogenates using the colorimetric method on spectrophotometer MARCEL S350 PRO (Marcel Sp. z o.o., Zielonka, Poland) according to the method described earlier [99] and expressed as μM of L-citrulline formation/g protein/min at 37 °C. The micromethod of Tain and Baylis [113] based on the rate of L-citrulline production was adopted to the macromethod. Shortly, homogenates of liver tissue were mixed with phosphate buffer (pH = 6.5). The samples were incubated at 37 °C for 45 min after the addition of 1 mM ADMA. The samples were centrifuged after the reaction had been stopped with 4% sulfosalicylic acid. Later, the oxime reagent (diacetyl monoxime (0.08% *w*/*v*) in 5% acetic acid) mixed with antipyrine/H_2_SO_4_ (antipyrine (0.5% *w*/*v*) in 50% sulfuric acid) reagent was added. Next, samples were once more incubated at 60 °C for 110 min and later cooled in an ice bath for 10 min. Serial dilutions of L-citrulline were used as standard. L-citrulline formation was measured at 466 nm. Values were subtracted from respective blanks (without ADMA).

### 4.9. Histological Evaluation

Part of the urinary bladders and one kidney were fixed in 4% buffered formalin and later prepared for histological evaluation by embedding in paraffin, cutting into 4 µm slices, and staining with hematoxylin and eosin. Samples were coded and assessed blindly by an experienced pathologist using a microscope Olympus BX41 (Olympus Corporation, Tokyo, Japan) with Fujitsu computer system (Fujitsu, Tokyo, Japan). In the kidney, the following features were determined for the scoring system: the presence of interstitial hemorrhages, glomerular atrophy, and dilation of urinary spaces. For each pathology, 0 points were given if it was absent or 1 point was given if it was present in a studied sample (total 3 points). In samples of urinary bladders, inflammatory infiltration, hyperemia, edema, ulcerations, erosions, and hemorrhages were determined for the scoring system. For each observed pathology, 0 points were given when absent and 1 point was given if present in the evaluated sample (total 6 points). From representative samples, the images are taken in 100× magnification.

### 4.10. Statistical Analysis

To express experimental data on all figures, means ± standard deviations (SD) were used. Statistical analysis of the effects of the substances on the studied parameters was carried out with the analysis of variance (ANOVA). The equality of variances was checked by the Brown–Forsythe test and specific comparisons between studied groups were later performed with Tukey’s post hoc test. In the case of inequality of variance (creatinine, hematuria, and histological urinary bladder score), the Kruskal–Wallis test was used as a post hoc test. Hypotheses were positively verified if *p* < 0.05. Statistical analysis was performed using TIBCO STATISTICA 13.3 PL (StatSoft, Kraków, Poland). Figure 2, Figure 3 and Figure 4 and Figure 6, Figure 7 and Figure 8 were performed using GraphPad Prism version 8.0 (GraphPad Software, San Diego, CA, USA). 

## 5. Conclusions

Administration of CPX caused many damages to the urinary bladder and kidneys. The best protection against CPX-induced injury was found to be mesna, which improved most of the studied parameters. Carvedilol failed to counteract the injury caused in the urinary bladders, but restored kidney function as expressed in serum potassium and creatinine levels, and exerted antioxidant and anti-inflammatory action. Additionally, carvedilol and mesna alone or given together prevented CPX-induced plasma ADMA elevation. It cannot be excluded that protective action of carvedilol may be better expressed after longer administration, e.g., 21–28 days, similarly to the duration of breaks between cycles of chemotherapy. The results of our study, along with the data published by other researchers, support the hypothesis of possible nephroprotection exerted by the third generation of beta adrenolytic drugs.

## Figures and Tables

**Table 1:** Statistical comparisons of hematuria measured semi-quantitatively on the dipstick test between studied groups. Mean values of hematuria in the studied time points with corresponding SD are presented in Figure 4. A detailed description of experimental groups is presented in Figure 1 and in the Materials and Methods section. * *p* < 0.05, *** *p* < 0.001 vs. CON group; ^##^ *p* < 0.01, ^###^ *p* < 0.001 vs. CPX group; ^ *p* < 0.05, ^^ *p* < 0.01, ^^^ *p* < 0.001 vs. CPX-C group.

	5 min before *i.p.* CPX or Normal Saline Injection	4 h after *i.p.* CPX or Normal Saline Injection	8 h after *i.p.* CPX or Normal Saline Injection	24 h after *i.p.* CPX or Normal Saline Injection	48 h after *i.p.* CPX or Normal Saline Injection
CON	0.0 ± 0.0	0.0 ± 0.0	0.0 ± 0.0	0.0 ± 0.0	0.0 ± 0.0
CPX	0.0 ± 0.0	2.58 ± 0.67 ***	3.92 ± 0.29 ***	4.00 ± 0.0 ***	4.00 ± 0.0 ***
CPX-M	0.0 ± 0.0	0.17 ± 0.25 ^###^	0.13 ± 0.31 ^###,^^^^	0.58 ± 0.63 ^###,^^	1.04 ± 1.03 ^###^
CPX-C	0.0 ± 0.0	1.29 ± 1.16 *	3.75 ± 0.62 ***	3.33 ± 0.65 ***	2.92 ± 0.29 ***
CPX-MC	0.0 ± 0.0	0.17 ± 0.39 ^###^	0.29 ± 0.45 ^##,^^^	0.42 ± 0.51 ^###,^^^	0.17 ± 0.39 ^###,^^^

## Data Availability

The data is contained in the article.

## Abbreviations

ADMA	Asymmetric dimethylarginine
ATP	Adenosine triphosphate
CAT	Catalase
CCl_4_	Carbon tetrachloride
CPX	Cyclophosphamide
COX	Cyclooxygenase
DDAH	Dimethylarginine dimethylaminotransferase
eNOS	Endothelial nitric oxide synthase
GSH	Glutathione
IL	Interleukin
iNOS	Inducible nitric oxide synthase
I/R	Ischemia reperfusion
MDA	Malondialdehyde
MDR	Multidrug resistance
MMP	Matrix metalloproteinases
NAD^+^	Oxidized form of nicotinamide adenine dinucleotide
NF-κB	Nuclear factor kappa B
NO	Nitric oxide
NS	Not significant
ONOO^–^	Peroxynitrite
PAF	Platelet-activating factor
PARP	Poly (ADP-ribose) polymerase
PGE_2_	Prostaglandin
PRMT	Protein arginine methyltransferase
ROS	Reactive oxygen species
SD	Standard deviation
SOD	Superoxide dismutase
TGF-β1	Transforming growth factor beta 1
TNF-α	Tumor necrosis factor alpha
